# Determinants of vascular impairment in type 1 diabetes–impact of sex and connexin 37 gene polymorphism: A cross-sectional study

**DOI:** 10.1186/s12933-024-02401-0

**Published:** 2024-08-22

**Authors:** Pavlína Piťhová, Michaela Cichrová, Milan Kvapil, Jaroslav A. Hubáček, Dana Dlouhá, Jan Piťha

**Affiliations:** 1 Department of Geriatric Internal Medicine, 2nd Medical Faculty Motol, Prague, Czech Republic; 2 Faculty of Mathematics and Physics, Charles University in Prague, Prague, Czech Republic; 3 Institute of Computer Science, Czech Academy of Sciences, Prague, Czech Republic; 4 Centre for Experimental Medicine, Institute for Clinical and Experimental Medicine, Prague, Czech Republic; 5 Department of Cardiology, Institute for Clinical and Experimental Medicine, Prague, Czech Republic

**Keywords:** Type 1 diabetes mellitus, Vascular parameters, Cardiovascular risk factors, Sex, Gene for connexin 37

## Abstract

**Background:**

The associations of risk factors with vascular impairment in type 1 diabetes patients seem more complex than that in type 2 diabetes patients. Therefore, we analyzed the associations between traditional and novel cardiovascular risk factors and vascular parameters in individuals with T1D and modifications of these associations according to sex and genetic factors.

**Methods:**

In a cross-sectional study, we analyzed the association of risk factors in T1D individuals younger than 65 years using vascular parameters, such as ankle brachial index (ABI) and toe brachial index (TBI), duplex ultrasound, measuring the presence of plaques in carotid and femoral arteries (Belcaro score) and intima media thickness of carotid arteries (CIMT). We also used photoplethysmography, which measured the interbranch index expressed as the Oliva-Roztocil index (ORI), and analyzed renal parameters, such as urine albumin/creatinine ratio (uACR) and glomerular filtration rate (GFR). We evaluated these associations using multivariate regression analysis, including interactions with sex and the gene for connexin 37 (Cx37) polymorphism (rs1764391).

**Results:**

In 235 men and 227 women (mean age 43.6 ± 13.6 years; mean duration of diabetes 22.1 ± 11.3 years), pulse pressure was strongly associated with unfavorable values of most of the vascular parameters under study (ABI, TBI, Belcaro scores, uACR and ORI), whereas plasma lipids, represented by remnant cholesterol (cholesterol – LDL-HDL cholesterol), the atherogenic index of plasma (log (triglycerides/HDL cholesterol) and Lp(a), were associated primarily with renal impairment (uACR, GFR and lipoprotein (a)). Plasma non-HDL cholesterol was not associated with any vascular parameter under study. In contrast to pulse pressure, the associations of lipid factors with kidney and vascular parameters were modified by sex and the Cx37 gene.

**Conclusion:**

In addition to known information, easily obtainable risk factor, such as pulse pressure, should be considered in individuals with T1D irrespective of sex and genetic background. The associations of plasma lipids with kidney function are complex and associated with sex and genetic factors. The decision of whether pulse pressure, remnant lipoproteins, Lp(a) and other determinants of vascular damage should become treatment targets in T1D should be based on the results of future clinical trials.

**Graphical Abstract:**

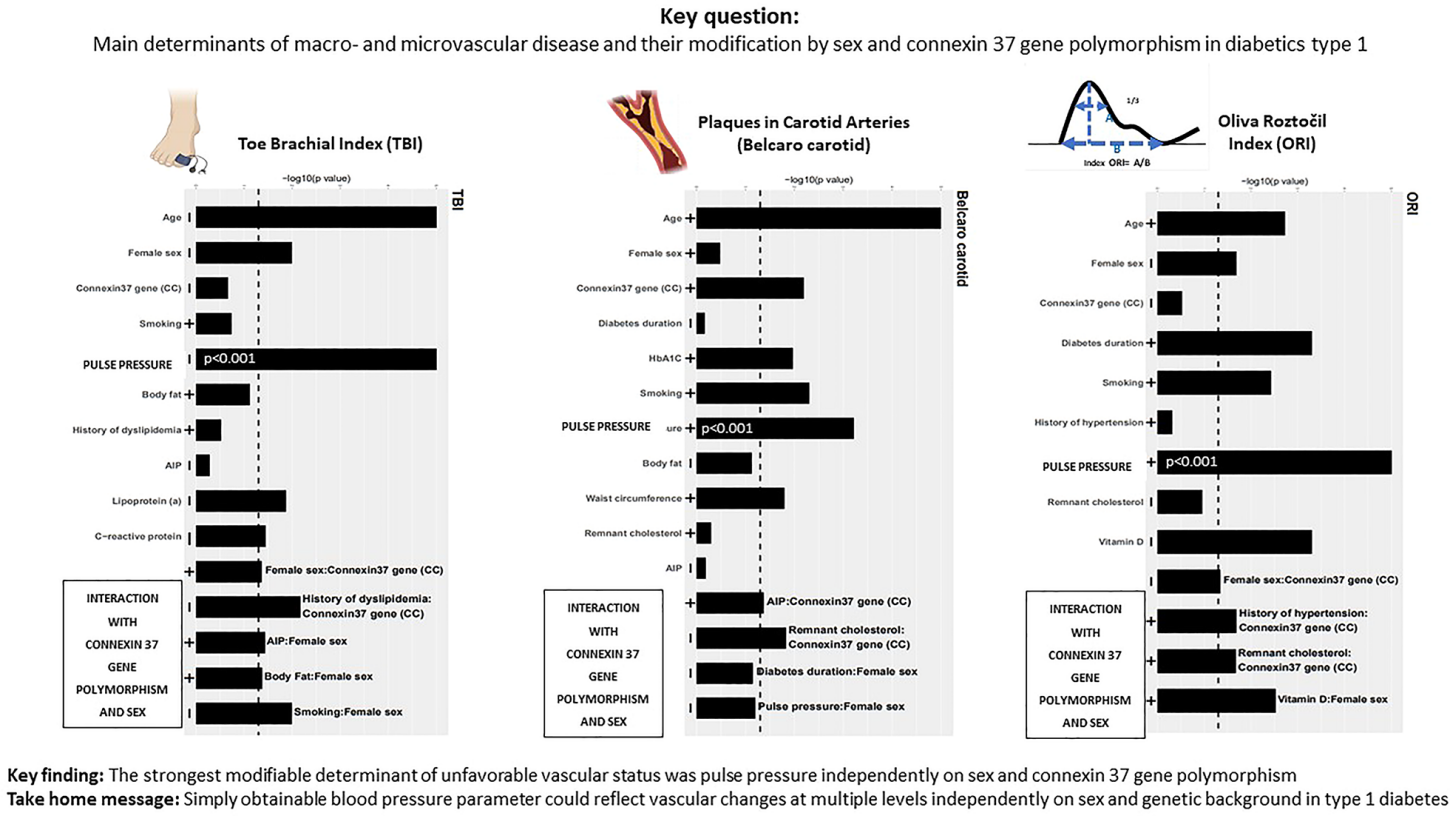

**Supplementary Information:**

The online version contains supplementary material available at 10.1186/s12933-024-02401-0.

## Background

Type 2 diabetes (T2D) and type 1 diabetes (T1D) [1] are among the most deleterious cardiovascular risk factors. While the mechanisms of vascular disease are relatively well described in T2D patients, the associations of non-glycemic risk factors with vascular impairment in T1D patients seem more complex and more difficult to assess [2]. Despite the documented decrease in cardiovascular mortality in T1D patients in some countries, the mortality in T1D patients is several times higher than the general population [3]. Therefore, cardiovascular prevention in T1D patients should not be based only on data from T2D patients. This presumption is also supported by our previous work in diabetic women, in which preclinical atherosclerosis, expressed as the intima‒media thickness of the common carotid and femoral arteries measured by high-resolution ultrasound, was strongly associated with factors reflecting body fat and its distribution in T1D women, whereas it was associated with factors reflecting primarily glucose and lipid disorders in T2D women [4]. Importantly, the process of vascular changes in T1D starts insidiously at a young age, but these changes clinically manifest later in life, and a wide range of metabolic and hemodynamic abnormalities directly, but also indirectly, associated with diabetic status may be responsible for vascular dysfunction and clinical events [5]. Therefore, the identification of major predictors of vascular abnormalities in their early stages could lead to better prevention. For predictors of cardiovascular events, metabolic control in T1D patients is critical in the prevention of cardiovascular disease [6]. However, the correction of other risk factors, including dyslipidemia, hypertension, and nephropathy, is also highly important. Therefore, better identification of additional non-glycemic predictors of cardiovascular disease may substantially improve therapeutic strategies in T1D patients. Vascular diseases may be detected by medical history and physical examination, but more sophisticated methods of detecting vascular changes using various non-invasive methodologies are available and used in people with diabetes, including early vascular changes in young individuals with T1D [7–10].

Genetic factors may also play important roles as predictors and/or modifiers of cardiovascular events in T1D patients. For example, the Joslin Diabetes Center 50-Year Medalist Study [11] showed that approximately 30–35% of individuals with T1D did not manifest significant microvascular complications over 50 years, regardless of their glycated hemoglobin (HbA1c) levels and traditional cardiovascular risk factors. These findings indicate that individuals suffering from T1D may possess a genetic background that accelerates or attenuates the adverse effects of metabolic and hemodynamic factors. In this respect, the role of the connexin 37 (Cx37) gene polymorphism as a potential candidate gene for cardiovascular disease has been evaluated and discussed. The C1019T polymorphism in the human connexin 37 gene (Cx37, encoded by GJA4) is associated with coronary artery disease [12], and its association with cardiovascular events seems strongly modified by the presence of T2D [13]. However, no data are available for T1D patients. This approach also offers some pathophysiological background on the impaired communication between cells in the vascular wall in people with diabetes. Our previous study revealed that the Cx37 gene was associated with subclinical atherosclerosis in women with type 1 and 2 diabetes and women with advanced central obesity, and the presence of the C allele indicated increased risk. In another study, we detected that the TT genotype of the Cx37 polymorphism was protective against subclinical atherosclerosis in women with higher fasting glycemia. These findings suggest that the Cx37 gene exerts completely different effects on the arterial wall depending on glycemia [12, 13]. Sex is another robust non-modifiable factor associated with cardiovascular disease. Data from a large meta-analysis indicated that females with T1D were at higher risk for fatal events than men [14]. Therefore, sex differences in cardiovascular risk profiles and the associations of cardiovascular risk factors with vessel impairment are extremely important.

Based on these findings, we evaluated the associations between multiple cardiovascular risk factors and a wide range of vascular parameters, including renal parameters, in middle-aged T1D patients. In addition, we evaluated potential modifications of those associations by sex and the Cx37 gene polymorphism.

## Methods

### Design of the study

The study was a cross-sectional observational study in unselected individuals with T1D followed in one center. The independent variables were traditional and novel cardiovascular risk factors, and the dependent variables were directly measured vascular parameters.

### Study population and procedures

All consecutive women and men with T1D, as defined by history and confirmed from medical records (all participants were followed for more than 20 years in one center), were examined. From 2012 to 2016, 227 women and from 2016 to 2020, 235 men were included in the study. The Institutional Review Board approved the study, and all participants signed informed consent forms. The investigation conformed to the principles outlined in the Declaration of Helsinki. The inclusion criteria were willingness to provide informed consent and age younger than 65 years. All participants provided informed consent before they were included in the study and after receiving comprehensive information. Histories were obtained using standardized questionnaires focused on the presence of cardiovascular disease of atherosclerotic origin, microvascular disease and cardiovascular risk factors. Anthropometric parameters and blood pressure were recorded during the physical examination. Fasting venous blood samples and urine samples were taken in the morning before examination and processed by a certified local laboratory.

### Cardiovascular risk factors, kidney vascular and genetic parameters

The duration of diabetes and the presence of macrovascular and microvascular disease were established on the basis of patient history and confirmed by medical reports. Participants who reported current or past regular smoking were defined as smokers. Hypertension and dyslipidemia were defined by current or previous treatment irrespective of the actual values of blood pressure and lipid concentration. Waist circumference was measured 5 cm above the umbilicus using paper tape while the patient was standing. The total body fat in % was measured using a hand-held body fat monitor (Omron BF306, Healthcare Co., Ltd., Matsusaka, Japan). Blood pressure and heart rate were obtained via three measurements using a mercury sphygmomanometer at intervals of 1 min in the seated position at the right upper extremity, and pulse pressure (the difference between the mean systolic and mean diastolic blood pressure obtained from the last two measurements) was subsequently analyzed. HbA1c was used as a parameter of metabolic control, and, the estimated glucose disposal rate (eGDR) was used as a parameter of insulin resistance and calculated as 24.31-(12.22 x WHR)-(3.29xHT)-(0.57xHbA1c), where WHR was the waist‒ hip ratio in cm, HT was the presence of hypertension, and HbA1c was glycated hemoglobin in mmol/mol [15].

Lipid parameters, including plasma non-HDL cholesterol (total cholesterol-HDL cholesterol), remnant cholesterol (RLPC) (total – LDL-HDL cholesterol), the atherogenic index of plasma (AIP) ((log (triglyceride/HDL cholesterol)) [16], and lipoprotein(a) ((Lp(a)) were measured using standard laboratory methods. Similarly, laboratory parameters, such as total leukocyte, neutrophil, lymphocyte, and platelet counts, C-reactive protein levels measured using the highly sensitive method (hsCRP), the glomerular filtration rate (GFR) calculated on the basis of the cystatin C concentration [17], and liver parameters, including gamma-glutamyl transferase (GGT), were obtained in a local laboratory via routine laboratory measurements. The fibrosis-4 liver index (FIB-4) was estimated as age *AST)/(platelet count* √^2^ALT) when age was expressed in years, ALT and AST were expressed in µkat/l, and the platelet count in 10^9^/L. Plasma vitamin D concentration was measured via chromatography. The urine albumin/creatinine ratio (uACR) was measured from spot urine samples that were collected during the study visits and processed in a local laboratory.

The Cx37 gene polymorphism (rs1764391) was established as previously described [12]. In brief, DNA was isolated from frozen EDTA-treated blood [18]. To genotype the C1019 > T (Pro319 > Ser) variant within the Cx37 gene, oligonucleotides 5` CTGGACCCACCCCCTCAGAATGGCCAAAGA and 5` AGGAAGCCGTAGTGCCTGGTGG and restriction enzyme AasI (Fermentas, Lithuania) were used to distinguish the T (fragments of 240 bp and 35 bp) and C (275 bp) alleles. A set of 24 samples was analyzed three times within 3 weeks with 100% conformity.

### Vascular parameters

The presence of cardiovascular disease was defined as cardiovascular disease of atherosclerotic origin (ischemic heart disease, ischemic stroke and clinically manifested peripheral artery disease), microvascular disease (retinopathy and nephropathy) and diabetic foot. In addition, directly measured vascular parameters were represented by ankle-brachial (ABI) and toe-brachial (TBI) indices, which were obtained as ratios of pressures at the ankles and toes using a sphygmomanometer and a photoplethysmographic probe (Hadeco Smartdop 50) for signal detection, divided by pressures at the upper extremities, which were measured with a regular sphygmomanometer. The ABI and TBI were automatically calculated by the software. The lesser known and less frequently used plethysmographic vascular parameter, Oliva-Roztocil interbranch index (ORI), was calculated from photoplethysmographic curves via software using the same photoplethysmographic probe (Hadeco Smartdop 50). This method was introduced several decades ago and is based on manual measurements of several rather simple parameters (height and contour) of pulse waves obtained via plethysmography, which is now computerized. The particular value for each patient was calculated as the distance measured between the ascending and descending arms of the pulse wave contour at two-thirds of the signal amplitude and was standardized for the heart rate period (Graphical Abstract). The value of ORI is considered normal if lower than 0.26 (without units). A higher value indicates a increased arterial stiffness and obstructive changes of smaller arteries in lower extremities. This parameter has been used in several studies for assessment of the cardiovascular system [19–21].

Changes of greater vessels were represented by carotid intima media thickness (CIMT) at the far walls of common carotid arteries twice on both sides at a distance of 1 cm from the bulb. The mean value, calculated from two values on both sides, was used for subsequent analysis. In addition to CIMT, we used a semiquantitative method based on the detection of focal changes called the Belcaro score of carotid arteries (BSCar) and femoral bifurcation (BSFem) [22]. This classification defines the degree of preclinical atherosclerosis on the basis of four ultrasound criteria. Class I represents three normal ultrasonic layers (intima media, adventitia, and periadventitia) clearly separated with no disruption of the lumen‒intima interface for at least 3.0 cm and/or initial alterations (lumen-intima interface disruption at intervals of < 0.5 cm). Class II represents intima-media granulation, granular echogenicity of the deep, normally anechoic intimal-medial layer and/or increased intima-media thickness (> 1 mm). Class III represents plaque without hemodynamic disturbance, localized wall thickening and increased density involving all ultrasonic layers, with an intima‒media thickness > 2 mm. Finally, Class IV represents plaque, as in Class III, but with stenosis on duplex scanning indicating stenosis > 50%. CIMT and Belcaro scores were measured using a duplex ultrasound device (Toshiba Nemio MX, Japan) with a Toshiba PLN-805AT linear array ultrasound transducer probe (frequency range of 6.0–12.0 MHz). All vascular measurements were performed after at least 5 min of rest in the supine position by a single experienced investigator (PP) during one session in a quiet room with a stable temperature of approximately 21 degrees Celsius.

### Statistical analyses

**Fig. 1:**
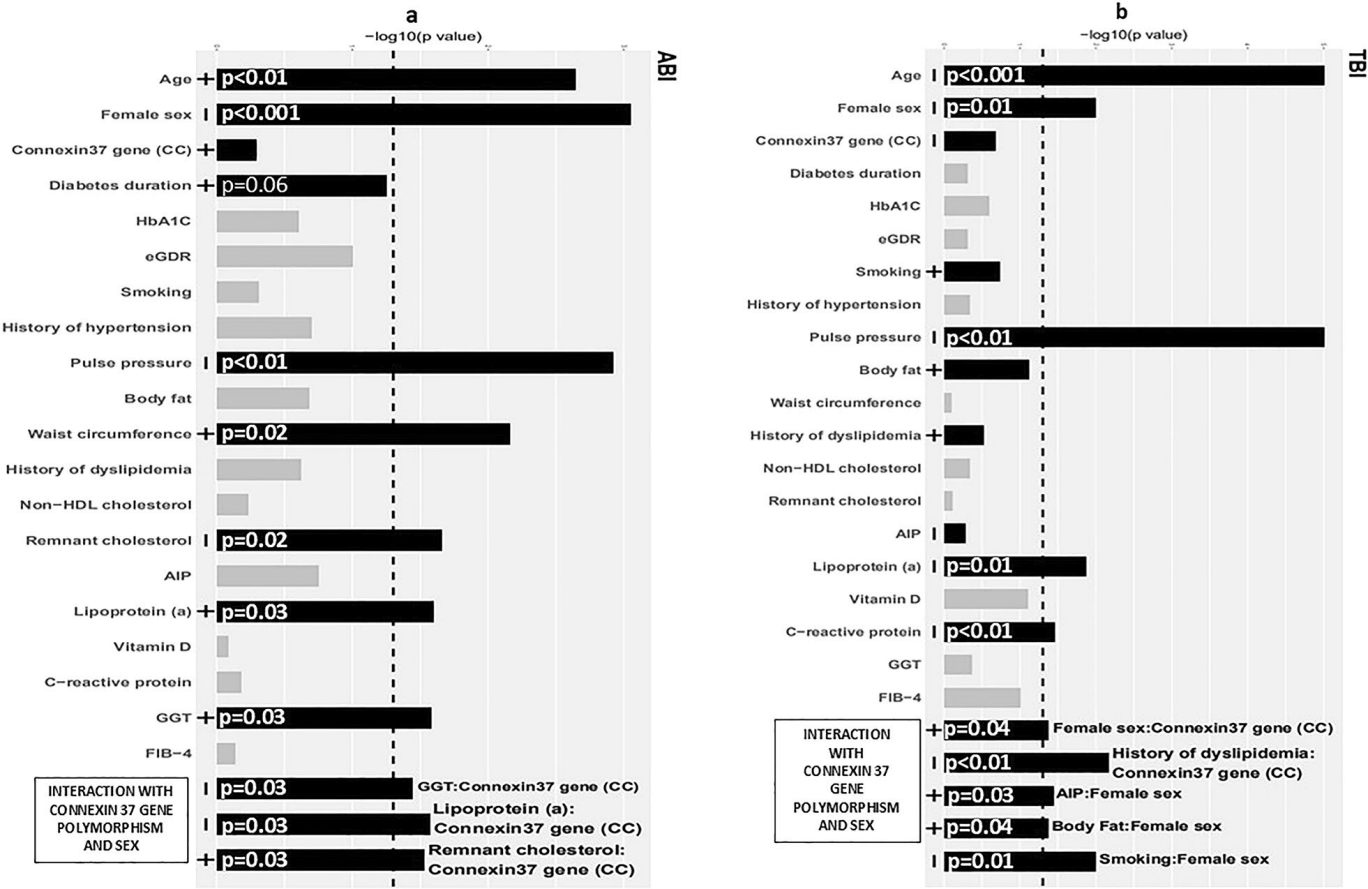
Determinants of the ankle brachial index (**a**) and toe brachial index (**b**) in type 1 diabetes patients Multivariate analysis (x axis **-** logarithmic transformation of p value/p values depicted as inverse logarithms: longer bars correspond to lower p values; black bars indicate significant associations and/or modifications by sex or connexin 37 gene polymorphism; gray bars: no association and no interaction; minus/plus signs at the basis of the bars indicate positive or inverse associations). Abbreviations: FIB-4-fibrosis-4 liver index; GGT-gamma-glutamyl transferase; HbA1c-glycated hemoglobin eGDR: Estimated glucose disposal rate; Connexin 37 gene (CC)-CC homozygotes

**Fig. 2:**
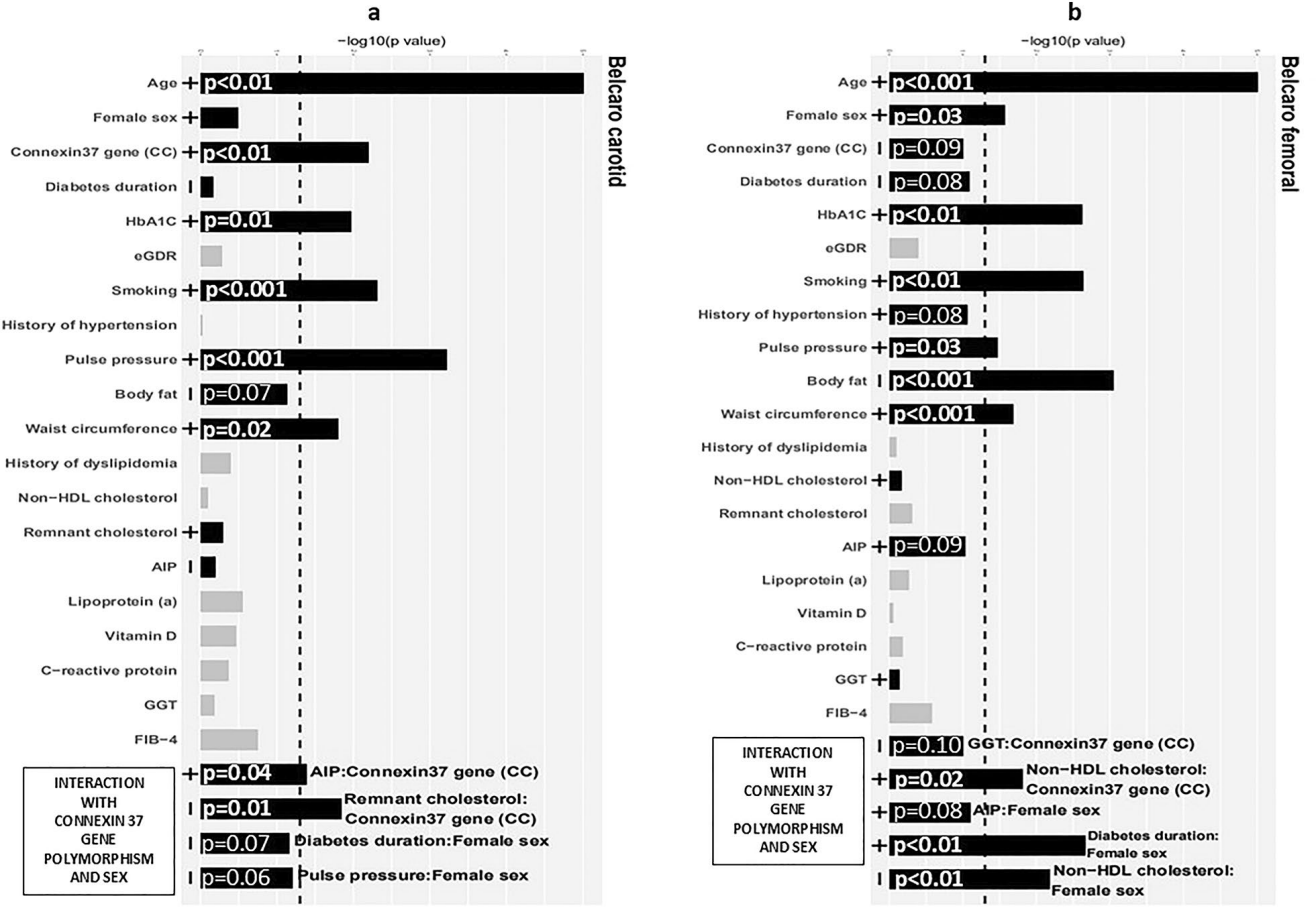
Determinants of the Belcaro score of the carotid (**a**) and femoral (**b**) arteries in type 1 diabetes patients (x-axis: p values depicted as inverse logarithms: longer bars correspond to lower p values; black bars indicate significant associations and/or modifications by sex or connexin 37 gene polymorphism; gray bars: no association and no interaction; minus/plus signs at the basis of the bars indicate positive or inverse associations). Abbreviations: FIB-4-fibrosis-4 liver index; GGT-gamma-glutamyl transferase; HbA1c-glycated hemoglobin,; eGDR- estimated glucose disposal rate; connexin 37 gene (CC)-CC homozygotes

**Fig. 3:**
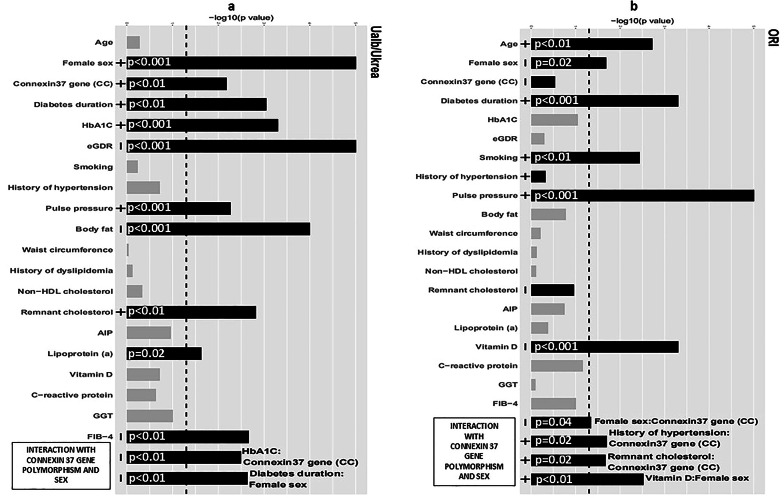
Determinants of the urine albumin/creatinine ratio (**a**) and Oliva-Roztocil index (**b**) in individuals with type 1 diabetes (x-axis: logarithmic transformation of p value/p values depicted as an inverse logarithm: longer bars correspond to lower p values; black bars indicate significant associations and/or modifications by sex or connexin37 gene polymorphism; gray bars: no association and no interaction; minus/plus signs at the basis of the bars indicate positive or inverse associations). Abbreviations: FIB-4 - fibrosis-4 liver index; GGT–gamma-glutamyl transferase; HbA1c–glycated hemoglobin; eGDR: estimated glucose disposal rate, ORI–Oliva-Roztocil Index; Connexin 37 gene (CC)–CC homozygotes

We used a wide range of multiple statistical approaches. The statistical analysis was performed using two different types of multivariate regression models that were selected according to the characteristics of the dependent variables; linear regression models based on ordinary least squares [23] were used for ABI, TBI, ORI, CIMT, GFR and uACR, and ordinal logistic regression via a cumulative link model [24] was used for Belcaro scores (3 groups were compared; in BSCar I vs. II vs. III-IV, in BSFem I, II vs. III vs. IV). The final models were constructed in a backward stepwise manner, and statistically insignificant variables were dropped in a hierarchical manner. The initial models incorporated all of the variables under study and their interactions with sex and the Cx37 gene polymorphism (CT/TT vs. CC). Through model comparisons and exploratory analyses, several continuous independent variables were transformed to more accurately represent their potential effects on the dependent variables. For example, hsCRP was categorized into intervals in all models (0.5, 0.5–1.5, 1.5–2.5, and > 2.5 mg/l). Other transformations of continuous independent variables and details are listed in the Supplementary material. The dependent variable, uACR, was log-transformed. For the final model, we evaluated the overall effect of the independent variables (simultaneously testing all coefficients corresponding to the independent variables—main effects and interactions) and the main effects and interactions separately, specifically for ABI, TBI, ORI, uACR, BSCar and BSFem. The corresponding p values are listed and displayed graphically as inverse logarithms (Figs. 1, 2 and 3 and Graphical Abstract). For the linear regression models, submodel F tests with heteroscedasticity-consistent standard errors [25, 26] were used in the model-building process and for testing the final model. For the ordinal logistic regression, p values for the model coefficients were calculated using Wald tests, and likelihood ratio tests were used during model development. The population differences for males and females were assessed using t tests and proportion tests. All statistical analyses were performed using R software [27, 28] at a 5% significance level, and no adjustments were made to account for multiple testing. A more detailed description of the transformations of particular continuous independent variables and more detailed analyses of individual variables are listed in the Supplementary material. Finally, because of multiple testing, we considered associations and interactions to be strong when *p* ≤ 0.01 and moderate when *p* > 0.01.

## Results

### Basic characteristics of the study population and sex differences

In total, 260 women and 260 men younger than 65 years with T1D were included. Complete data for the purpose of this study were obtained from 227 men and 235 women (mean age 43.6 ± 13.6 years; mean duration of diabetes 22.1 ± 11.3 years). As shown in Table 1, women and men did not differ in the mean age or mean duration of diabetes, but women had higher HbA1c levels than men, and women had higher eGDR than men. Men reported smoking and a history of hypertension more often than women. Women and men reported similar frequencies of dyslipidemia and cardiovascular and microvascular disease. No differences in the prevalence of the polymorphism of the Cx37 gene were detected between women and men. Body mass index and waist circumference were greater in men, and total body fat content was greater in women. Men had higher systolic and diastolic pressures than women but the pulse pressure did not differ between women and men. The AIP was higher (i.e., less favorable) in men, but there were no differences in the other lipid parameters under study. Men had higher values of vitamin D and GGT than women, and no differences were detected for hsCRP or FIB-4.

**Table 1:** Demographics and history data (all data are means ± SDs, if not stated otherwise).

	Women (*n* = 227)	Men (*n* = 235)	*p*
Age (years)	43.8 ± 12.7	43.3 ± 14.4	0.693
Duration of diabetes (years)	21.7 ± 10.2	22.5 ± 12.3	0.448
Smoking (%)	31.9%	43%	0.011
Hypertension (%)	37.9%	49.8%	< 0.001
Dyslipidemia (%)	30.1%	34.9%	0.275
Gene for connexin 37 n (%) TT/CT/CC genotype	21/104/102 (9.3/45.8/44.9)	24/101/110 (10.2/43.0/46.8)	0.816
ASCVD *n* (%)	21 (9.2)	30 (12.7)	0.239
Retinopathy/treatment *n* (%)	111/47 (48.8/20.8)	115/54 (48.9/23.1)	1.000/0.575
Nephropathy *n* (%)	65 (28.6)	73 (30.6)	0.612
Diabetic foot *n* (%)	17 (7.4)	27 (11.5)	0.156
Body mass index (kg.m^−2^)	25.4 ± 4.5	26.9 ± 4.2	< 0.001
Waist circumference (cm)	80.2 ± 10.7	92.4 ± 12.0	< 0.001
Total body fat (%)	27.9 ± 7.3	19.29 ± 7.6	< 0.001
Systolic blood pressure (mm Hg)	127.4 ± 17.0	132.5 ± 17.5	< 0.001
Diastolic blood pressure (mm Hg)	73.6 ± 9.6	76.7 ± 9.2	< 0.001
Pulse pressure (mm Hg)	53.2 ± 13.4	55.7 ± 14.4	0.054
Non-HDL cholesterol (mmol/l)	3.18 ± 0.85	3.33 ± 0.91	0.068
Atherogenic index of plasma	−0.27 ± 0.26	−0.09 ± 0.27	< 0.001
Remnant cholesterol (mmol/l)	0.56 ± 0.37	0.60 ± 0.43	0.285
Lipoprotein (a) (mg/l)	254.1 ± 369.6	251.2 ± 332.1	0.929
hsCRP (mg/l)	2.21 ± 2.25	2.19 ± 3.29	0.939
HbA1c (mmol/mol)	68.1 ± 14.8	62.7 ± 14.6	< 0.001
eGDR	8.36 ± 2.31	7.03 ± 2.36	*p* < 0.001
Plasma vitamin D (nmol/l)	53.39 ± 27.17	58.12 ± 22.1	0.05
Gamma-glutamyl transferase (ukat/l)	0.38 ± 0.35	0.63 ± 0.84	< 0.001
FIB-4	0.87 ± 0.49	0.98 ± 0.73	0.059
Ankle brachial index	1.04 ± 0.11	1.11 ± 0.18	< 0.001
Toe brachial index	0.8 ± 0.12	0.86 ± 0.14	< 0.001
CIMT (mm)	0.69 ± 0.16	0.76 ± 0.18	< 0.001
Belcaro score carotid I/II/III/IV n (%)	141/79/6/1 (61.7/35.2/2.6/0.004)	103/103/27/2 (43.8/43.8/11.5/0.008)	< 0.001
Belcaro score femoral I/II/III/IV n (%)	166/37/24/0 (72.7/16.3/10.6/0)	136/67/31/1 (57.9/28.5/13.2/0.004)	< 0.001
Oliva-Roztocil index	0.27 ± 0.04	0.26 ± 0.04	< 0.001
Urine albumin/creatinine (mg/mol creatinine); mean (95% CIs)	7.82 (2.96, 2.68)	17.3 (9.34, 25.26)	0.049
Glomerular filtration rate (ml/s/1.73m^2^)	1.80 ± 0.65	1.49 ± 0.42	< 0.001

*ASCVD* Cardiovascular disease of atherosclerotic origin; *CIMT* Intima-media thickness of the common carotid artery; *eGDR* Estimated glucose disposal rate: 24.31-(12.22xWHR)-(3.29xHT)-(0.57xHbA1c); *FIB-4* Fibrosis 4 liver index; *hsCRP *C-reactive protein measured using the high sensitivity method.

For the vascular parameters, women had lower ABI, TBI and higher ORI (less favorable values) than men, whereas men had higher CIMT, BSCar, BSFem and uACR and lower cystatin C clearance than women.

For the Cx37 gene Hardy-Weinberg equilibrium, the p values for both groups under study were 0.45 and 0.91, and it was 0.51 for the whole group.

All participants were treated with intensified insulin therapy; 49% of the women and 43% of the men used insulin pump therapy. In addition, 25% of women and 31% of men used continuous glucose sensors or flash glucose monitoring regularly during the previous year. A total of 8% of women and 6% of men needed additional metformin therapy to improve their insulin sensitivity. Men were treated with a higher mean insulin dose (0.66 ± 0.19 vs. 0.62 ± 0.22 IU/kg/day, *p* < 0.01) and were more frequently treated with ACE/ARB1 inhibitors than women (46% vs. 35%, *p* < 0.01), but no significant difference was observed for hypolipidemic treatment (24% vs. 29%). In addition, 30% of women reported menopausal status.

### Determinants of directly measured vascular and kidney vascular parameters

ABI (Fig. 1a) was positively and strongly associated with age, moderately associated with waist circumference, Lp(a) lower than 600 mg/l, and GGT (*p* < 0.001, *p* = 0.029, *p* = 0.025, and *p* = 0.026 for GGT equal to or greater than 0.65 ukat/l, respectively), and it was inversely and strongly associated with female sex and pulse pressure and inversely and moderately associated with RLPC (*p* = 0.003, *p* < 0.001, and *p* = 0.022, respectively). No modifying effect of sex was observed. For the modifying effect of the Cx37 gene polymorphism, ABI was positively and moderately associated with RLPC in CC homozygotes (*p* = 0.03), inversely and moderately associated with GGT (*p* = 0.032 for GGT equal to or greater than 0.65 ukat/l, respectively), and inversely and moderately associated with Lp(a) (*p* = 0.027). More detailed data are available in the Supplementary material (Tables 2 and 3).

TBI (Fig. 1b) was inversely and strongly associated with age, female sex, pulse pressure, and Lp(a) (*p* < 0.001, *p* = 0.01, *p* < 0.001, and *p* = 0.01, respectively), and it was inversely and moderately associated with hsCRP (*p* = 0.035, with individual coefficients *p* = 0.005–0.49, with the highest predicted values for hsCRP 0.5, followed by 1.5–2.5 and 0.5–1.5, respectively). For the modifying effect of sex, TBI was positively and moderately associated with higher body fat and higher AIP in females and inversely and strongly associated with a history of smoking (*p* = 0.029 and *p* = 0.04, and *p* = 0.01, respectively). For the modifying effect of the Cx37 gene, TBI was inversely and strongly associated with reported dyslipidemia in CC homozygotes (*p* = 0.007) and positively and moderately associated with female sex (*p* = 0.04). More detailed data are available in the Supplementary material (Tables 4 and 5).

BSCar (Fig. 2a) was strongly and positively associated with age, CC homozygosity, HbA1c, history of smoking, pulse pressure, and waist circumference (*p* < 0.001, *p* = 0.006, *p* = 0.01, *p* < 0.001, *p* < 0.001, and *p* = 0.002, respectively). No modifying effect of sex was observed. For the modifying effect of the Cx37 gene polymorphism, BSCar was inversely and strongly associated with RLPC in CC homozygotes and positively and moderately associated with AIP (*p* = 0.01 and *p* = 0.04, respectively) BSFem (Fig. 2b) was positively and strongly associated with age, HbA1c, smoking status and waist circumference and moderately associated with female sex and pulse pressure (*p* < 0.001, *p* = 0.002, *p* = 0.002, *p* < 0.001, *p* = 0.03 and *p* = 0.03, respectively), and it was inversely and strongly associated with body fat (*p* < 0.001). For the effect of sex, BSFem was inversely and strongly associated with non-HDL cholesterol in females and positively and strongly associated with diabetes duration (*p* = 0.007 and *p* = 0.002, respectively). For the modifying effect of the Cx37 gene polymorphism, BSFem was positively and moderately associated with non-HDL cholesterol in CC homozygotes (*p* = 0.02). More detailed data are available in the Supplementary material (Tables 6 and 7).

CIMT was positively and strongly associated with age and non-HDL cholesterol and moderately associated with diabetes duration and HbA1c (*p* < 0.001 and *p* = 0.007 and *p* = 0.02 and *p* = 0.02, respectively), and it was inversely and strongly associated with plasma vitamin D (*p* = 0.009). For the modifying effect of sex, it was positively and moderately associated with Lp(a) (*p* = 0.03) in females and inversely and moderately associated with non-HDL cholesterol (*p* = 0.05). No modifying effect of the Cx37 gene polymorphism was detected The uACR (Fig. 3a) was positively and strongly associated with female sex, duration of diabetes, HbA1c, history of hypertension, pulse pressure, AIP and FIB-4 and moderately associated with RLPC, Lp(a) and CC homozygosity (*p* = 0.003, *p* = 0.01, *p* < 0.001, *p* < 0.001, *p* = 0.004 *p* < 0.001 and *p* = 0.01, and *p* = 0.02, *p* = 0.02 and *p* = 0.02, respectively), and it was inversely and strongly associated with body fat (*p* < 0.001). For the modifying effect of sex, uACR was inversely and strongly associated with the duration of diabetes in females (*p* = 0.01). For the modifying effect of the Cx37 gene polymorphism, the uACR was inversely and strongly associated with HbA1c in CC homozygotes (*p* = 0.01) The ORI (Fig. 3b) was positively and strongly associated with age, diabetes duration, smoking, and pulse pressure (*p* = 0.002, *p* < 0.001, *p* = 0.004, and *p* < 0.001, respectively), and it was inversely and moderately associated with female sex and strongly associated with plasma vitamin D (*p* = 0.02 and *p* < 0.001., respectively). For the modifying effect of sex, ORI was positively and strongly associated with plasma vitamin D in females (*p* = 0.003). For the modifying effect of the Cx37 gene, ORI was positively and moderately associated with a history of hypertension and RLPC in CC homozygotes (*p* = 0.02 and *p* = 0.02, respectively) and inversely and moderately associated with female sex (*p* = 0.05). More detailed data are available in the Supplementary material (Tables 8 and 9).

The GFR was inversely and strongly associated with AIP, Lp(a), and hypertension and moderately associated with pulse pressure and smoking (*p* < 0.001, *p* = 0.01, *p* = 0.01, *p* = 0.02 and *p* = 0.02, respectively), and it was positively and moderately associated with age (*p* = 0.05). For the modifying effect of sex, the GFR was positively and moderately associated with age in females (*p* = 0.02). For the modifying effect of the Cx37 gene, the GFR was positively and strongly associated with the AIP in CC homozygotes (*p* = 0.01) Finally, for the intercorrelations between continuous vascular parameters (ABI, TBI, uACR, and ORI), an inverse strong linear correlation was found only between TBI and ORI (*r *= −0.46)

### Discussion

 In this study we detected significant associations between vascular and a wide range of cardiovascular risk factors representing traditional and newer factors. We also evaluated potential modifications of these associations by sex and Cx37 gene polymorphism. Although large prospective studies are available that focus on the effects of traditional risk factors [29], we consider our approach unique by studying the impact of multiple cardiovascular risk factors on vascular parameters ranging from parameters representing territories of greater vessels to parameters representing smaller vessels.

### Associations of vascular parameters with age, duration, control of T1D and insulin resistance

 For changes of greater vessels, age was strongly associated with unfavorable parameters, as expected, with the exception being albuminuria, whereas the duration of diabetes was strongly associated with parameters representing changes in smaller vessels, such as albuminuria and ORI, but not with vascular parameters representing changes in greater vessels. Therefore, these data indicate that hyperglycemia in T1D patients first affects smaller vessels. Changes of greater vessels, represented by plaques in the carotid and femoral arteries, but also more subtle vascular and/or changes in the kidney vasculature, are associated with metabolic control of diabetes, which supports the importance of glycemia control in the prevention of changes in all arterial territories in T1D patients For the parameters of insulin resistance, eGDR was associated with albuminuria, whereas waist circumference was strongly associated with atherosclerotic changes in the carotid and femoral arteries. However, this finding for eGDR was very likely modified by the presence of hypertension, which is included in the eGDR formula. Therefore, waist circumference should be proposed as a reliable marker for the assessment of vascular changes in T1D patients. The fact that total body fat was inversely associated with femoral atherosclerosis and albuminuria indicates its putative protective vascular effect in T1D. In women, this protective effect was observed in smaller vessels, as represented by TBI. However, if this protection also applies for obese people with diabetes (participants in this study had a normal body mass index), it is less probable, and because our study was cross-sectional, other factors beyond body fat may be responsible for this phenomenon. In addition to waist circumference, plasma triglycerides and triglyceride-rich lipoproteins may be considered potential indicators of insulin resistance [30] and are discussed in the following section.

### Associations of vascular parameters with smoking, blood pressure and plasma lipids

 The vascular changes represented by changes in carotid and femoral arteries (Belcaro score) and changes in smaller vessels and arterial stiffness represented by ORI were associated with smoking. Surprisingly, ABI, TBI and albuminuria were not associated with smoking in the whole group, and smoking was strongly associated with TBI only in women. This finding indicates that smoking preferentially affects smaller vessels in women with T1D. Interestingly, less favorable values of most vascular parameters under study, including parameters of kidney vascular function (with the exception of subtle vascular changes expressed as CIMT), were strongly associated with pulse pressure (Figs. 1, 2 and 3; Tables 2, 3, 4, 5, 6, 7 and 8 and Graphical Abstract).

These findings indicate that pulse pressure, even within the range of physiological values (Table 1), may be associated with vascular damage at multiple levels in T1D patients. The role of blood pressure in T1D was described in a large meta-analysis in which hypertension partially mediated the causal effects of T1D on peripheral and coronary atherosclerosis [5]. The pathophysiological explanation is that pulse pressure also reflects arterial stiffening in people with diabetes, and it is higher in T1D patients than non-diabetic population in a cross-sectional, case‒control study of almost 3,000 individuals. Although higher pulse pressure in this study was more pronounced in individuals with diabetic nephropathy, it was also found in participants with a normal albumin excretion rate compared to controls [31]. In another study, pulse pressure was used as the main endpoint in T1D [32]. In another study it was strongly associated with future ASCVD and also with microvascular disease including albuminuria in younger population of individuals with T1D in cross-sectional analysis [33]; in this study pulse pressure was also in the range of physiological values. In our study, the associations of pulse pressure with vascular parameters were not affected by sex or the Cx37 gene polymorphism. Therefore, increased pulse pressure as an indicator of future cardiovascular events in older populations [34] should also be considered in middle-aged people with T1D, irrespective of sex and genetic background.

In our study, the RLPC, which represents remnant lipoproteins, and the AIP, which represents reverse cholesterol transport, were strongly associated with albuminuria. The association of the AIP with lower cystatin C clearance further supports the potential interconnection of particular lipid parameters with kidney impairment. Furthermore, the association of RLPC with a lower ABI indicates a potentially greater impact of remnant lipoproteins on lower extremity arteries. In contrast to less frequently used lipid parameters, non-HDL cholesterol, which represents atherogenic lipoproteins, was not associated with any vascular parameter under study, whereas Lp(a), an emerging atherogenic lipid factor for intervention [35], was strongly associated with impaired kidney parameters, but it was also positively associated with ABI. Therefore, we speculate that Lp(a) may be associated with increased arterial stiffness, which was represented by a relatively higher ABI. Moreover, the latter association was moderate (*p* = 0.03), and only chance observation could not be excluded.

For the kidney parameters, similar results as remnant lipoproteins and reverse cholesterol transport were obtained for Lp(a), whereas no effect of atherogenic non-HDL cholesterol on kidney parameters was detected. These results may indicate a potential connection between specific lipid factors and kidney impairment. However, because of the cross-sectional nature of the study, we cannot exclude reverse associations between lipids and kidney parameters, as discussed in the literature [36]. However, the effects of traditional cardiovascular risk factors on kidney parameters were described in a prospective study in almost 28,000 children and adolescents with T1D. The risk factors for albuminuria in this study were diabetes duration, HbA1c, blood pressure, and LDL cholesterol, the latter of which was not confirmed in our study. The difference in the assessment of the effects of atherogenic lipids may be attributed to the different characteristics of the populations under study. Our study recruited older participants. Moreover, in the cross-sectional analysis in this prospective study, diabetes duration, HbA1c, dyslipidemia, blood pressure, and male sex, but not lipids, were identified as risk factors for nephropathy, after accounting for other cardiovascular risk factors [37]. In general, the exact role of lipid parameters in T1D has not been fully elucidated [38], and one of the reasons may be the close association of lipids with the metabolic control of T1D in contrast to the non-diabetic population [39].

### Novel risk factors

Most of the vascular markers under study were not strongly associated with novel risk factors, including inflammatory markers, such as plasma vitamin D and hsCRP, and markers of liver impairment, such as GGT and FIB-4. The only exception was associations of less favorable values of CIMT and ORI with vitamin D concentration, which was more pronounced in women. Vitamin D deficiency is considered a risk marker for major adverse cardiovascular events in T1D patients but not for microvascular complications or all-cause mortality [40]. On the basis of our data, unfavorable vascular changes may be associated with lower plasma vitamin D exclusively in women.

Inflammatory factors have been proposed as among the strongest nonlipid determinants of vascular damage [41]. In individuals with T1D, inflammatory markers were associated with carotid atherosclerosis in a cross-sectional study from Spain by Mariaca et al., which included a study population of comparable size and age as our study [42]. In contrast to the data of Mariaca et al., hsCRP in our study was associated with the impairment of smaller vessels, as represented by a lower TBI, but not with atherosclerosis in the carotid and femoral arteries. The explanation for this difference is that our population was less selective, and participants with durations of T1D less than 10 years were included, and fewer than 3% of the women and 12% of the men had detectable carotid plaques (BSCar > II) compared to 41% in Mariaca et al. However, the TBI was only moderately associated with hsCRP, with a predominantly negative trend (*p* = 0.035, with individual coefficients *p* = 0.005–0.49 and with the highest predicted values for hsCRP 0.5, followed by 1.5–2.5 and 0.5–1.5, respectively), which indicated a more complex association of this inflammatory factor with vascular disease.

GGT is considered another risk factor, especially for microvascular disease [43], and it was associated with a lower ABI representing rather impairment of greater vessels. However, GGT measurement via standard laboratory methods may miss particular GGT isoenzymes, which could play different roles in metabolic diseases [44]. Finally, FIB-4, a marker of liver fibrosis, insulin resistance, and cardiovascular disease in T1D [45] was associated primarily with albuminuria in our study. This finding highlights the potential role of non-glycemic and non-lipid factors in vascular and kidney disease.

### Sex and Cx37 gene polymorphism modifications for the observed associations

In women, we observed an inverse association of diabetes duration with albuminuria, whereas the association of diabetes duration with atherosclerosis in the femoral arteries was further enhanced. Moreover, in women, higher non-HDL cholesterol was associated with less femoral atherosclerosis. These findings indicate a potential protection of kidney function and arteries in the lower extremities against long-lasting glycemia and lipid factors in women. However, the effect of long-lasting glycemia on lower extremity arteries may be accelerated in women. In addition, the more deleterious effect of smoking on smaller arteries in women was described in one of the previous sections of this paper. We are not able to reliably propose mechanisms responsible for these findings because of the cross-sectional design of our study. In the literature, the proposed mechanisms of between-sex differences are based on psychosocial and biological factors. Psychosocial factors, including behavioral patterns and differences in treatment strategies, are difficult to characterize precisely. For the biological mechanisms, an imbalance of sex hormones in women with T1D may contribute to a more atherogenic lipid profile, insulin resistance, greater inflammation, and loss of the vasoprotective effect of the female sex observed at the epidemiological level in a non-diabetic population. However, unequivocal data are not presented. On the one hand, lower levels of estradiol in adolescents with T1D were proposed as a potential cause of increased risk in women compared to nondiabetic control women [46]. Among premenopausal women with diabetes, hypothalamic hypoestrogenism was more prevalent and associated with coronary artery disease [47]. On the other hand, a higher concentration of estradiol was proposed to be associated with increased vascular damage in T1D women [48]. Sex differences were also considered in the Steno type 1 risk engine to estimate the 10-year risk of developing cardiovascular events in a multicenter, cross-sectional study involving 2,041 middle-aged participants with T1D, including 45% women. In this study, the 10-year estimated cardiovascular risk was higher in men younger than 55 years than in women of similar age, and sex differences disappeared at ages ≥ 55 years. Therefore, sex differences may be modified by age, and as suggested by the authors, female sex is no longer protective around menopause. Therefore, in our study, attenuation or acceleration of the impact of particular risk factors on different vascular territories may also reflect the different hormonal statuses of T1D women. Interestingly, the self-reported frequency of macro- and microvascular disease was similar in women and men, whereas directly measured vascular parameters markedly differed between women and men.

For the Cx37 gene polymorphism, we observed that CC homozygosity may attenuate or even reverse the effect of metabolic control of diabetes (HbA1c) on albuminuria, but it accelerates the effect of remnant lipoproteins on carotid atherosclerosis. In addition, dyslipidemia in CC homozygotes was associated with lower values of TBI, but higher values of remnant lipoproteins, as represented by RLPC, were associated with fewer atherosclerotic changes in carotid arteries in the same group. This finding indicates a potential effect of the CC genotype on risk factor-vascular associations in individuals with T1D. Carriers of T or C allele of the Cx37 gene are at increased or decreased cardiovascular risk depending on the presence of diabetes, namely, T2D. Our data indicate a potential modifying effect of this gene in T1D. From a pathophysiological perspective, we speculate that the sensitivity of connexin 37 produced by different gene variants could differ in susceptibility to glycation, and this phenomenon could affect communication between cells in the vessel wall [49, 50]. However, potential modification of the functionality of Cx37 by hyperglycemia remains hypothetical.

### Strengths and limitations

 The main strength of the study is that it focused on an unselected population of thoroughly examined individuals with T1D followed in one center in a standard manner. In addition, we used several methods of detecting vascular disease, including less frequently used parameters, to assess the impact of a wide range of cardiovascular risk factors on the vasculature. For the hemodynamic risk factors, we focused on pulse pressure as an indicator of blood pressure because it also reflects arterial stiffness and may be an early and simple parameter for risk assessment in people with diabetes. In addition, we focused on the effects of non-LDL lipid parameters on the vascular system. In particular, we studied non-HDL cholesterol rather than LDL cholesterol (the correlation between both parameters was 0.89) and focused on representatives of triglyceride-rich lipoproteins [51] and Lp(a) [52], which are important risk factors in people with diabetes.

The main limitation of our study is its cross-sectional design, which does not allow us to definitely establish cause and effect relationships. However, because multiple factors and vascular parameters were studied with similar results, we consider most of the observed associations valid. Another limitation is that our findings are based on preclinical vascular parameters, which are not always associated with clinical events. However, as demonstrated in previous studies, most of the studied vascular parameters reflect the future risk of cardiovascular and often fatal events, and individuals with T1D are not exceptional in this respect. Although the connexin 37 gene was proposed as a candidate gene for ischemic heart disease, it represents a simple single nucleotide polymorphism, and more extensive and sophisticated genetic markers, including genome-wide associations, are now being studied. However, this particular gene marker has been intensively studied, and its potential association with ischemic heart disease has been repeatedly described, including potential modifications by diabetes and central obesity [12, 13, 53]. Finally, another limitation is that only the Caucasian population was studied, and we should be cautious when generalizing these results to other ethnic groups.

### Conclusion

This cross-sectional study confirms previously described findings in T1D. However, to the best of our knowledge, it also reveals new associations of blood pressure and lipids with vascular parameters and the modification of these associations by sex and genetic factors. In particular, we demonstrated that pulse pressure in middle-aged people with T1D was consistently associated with less favorable values for most vascular parameters, independent of sex and genetic factors. In our study, pulse pressure was associated with Cx37 gene polymorphism and lipid parameters were strongly associated with kidney vascular impairment and were modified by sex and Cx37 gene polymorphism. Therefore, easily obtainable parameters, such as pulse pressure, should be considered in individuals with T1D irrespective of sex and genetic background. The association of plasma lipids with kidney function is more complex, and we may have detected inverse relationships because of the design of our study. The decision of whether pulse pressure, remnant lipoproteins, Lp(a) and other determinants of vascular damage should become treatment targets in T1D should be based on the results of future clinical trials

### Electronic supplementary material

Supplementary Material 1

## Data Availability

No datasets were generated or analysed during the current study.

## Abbreviations

ABI: Ankle brachial index
AIP: Atherogenic index of plasma
ALT: Alanine-amino transferase
ASCVD: Cardiovascular disease of atherosclerotic origin
AST: Aspartate-amino transferase
BSCar: Belcaro score carotid
BSFem: Belcaro score femoral
Cx37: Connexin 37
CIMT: Carotid intima–media thickness of the common carotid artery
eGDR: Estimated glucose disposal rate
FIB-4: Fibrosis-4 liver index
GGT: Gamma-glutamyl transferase
HbA1c: Glycated hemoglobin
hsCRP: C-reactive protein measured by the high-sensitivity method
LDL cholesterol: Low-density cholesterol
Lp(a): Lipoprotein (a)
Non-HDL cholesterol: Total cholesterol – HDL cholesterol
ORI: Oliva-Roztocil interbranch index
RLPC: Remnant plasma cholesterol (total – LDL-HDL cholesterol)
TBI: Toe brachial index
T1D: Type 1 diabetes
uACR: Urine albumin/creatinine ratio
