# Supplementary material for: Determinants of vascular impairment in type 1 diabetes–impact of sex and connexin 37 gene polymorphism: A cross-sectional study
Source: Cardiovasc Diabetol. 2024 Aug 22;23:309. doi: 10.1186/s12933-024-02401-0 (PMC11342627; doi:10.1186/s12933-024-02401-0)
Supplement: Supplementary file 1 — Supplementary Material 1 [file 12933_2024_2401_MOESM1_ESM.docx]

**Supplementary information:**

**METHODOLOGY:**

**Statistical analyses:**

In all analyses, hsCRP was categorized (with cut-off values 0.5, 2.1, and 4 mg/L), the non-listed continuous independent variables were modelled linearly.

For TBI, body fat percentage and lipoprotein (a) (Lp(a)) were modelled logarithmically. For ORI, vitamin D and body fat was modelled logarithmically, and T1D duration quadratically. For ABI, the following transformations were utilized: FIB-4 was log-transformed; RLPC, HbA1c, and Lp(a) were modelled quadratically; waist circumference, pulse pressure, vitamin D, and body fat as piecewise linear with specified breakpoints, and GGT were categorized. Specifically, waist circumference was segmented at 85 cm, pulse pressure at 55 and 70 mmHg, vitamin D at 80 nmol/L, body fat at 29%, GGT at 0.3 and 0.65 ukat/L.

AIP and age were modeled quadratically, and vitamin D was categorized into 10-30, 30-65, 65+ nmol/L. For nephropathy, T1D duration, vitamin D, age, HbA1c, and GGT were logarithmically transformed, and non-HDL cholesterol was modelled quadratically. GGT and Lp(a) were logarithmically transformed.

Cystatin C clearance, waist circumference, Lp(a), and age were modelled logarithmically. For CIMT, GGT was logarithmically transformed, and T1D duration was modelled quadratically. For uACR it was necessary to logarithmically transform the response, additionally, pulse pressure was modelled quadratically, the effects of remnant LDL cholesterol (< 0.65, 0.65+ mg/dL), non-HDL cholesterol (< 3.3, 3.3+ mg/dl), and FIB-4 (< 1.5, 1.5+), were modelled using piecewise linear associations, and Lp(a) was categorized (0–100, 100–200, 200+ nmol/l). The BSFem score was categorized into three groups (1 - 2, 3, 4 ) to accommodate the distribution of observations. For the parameterization of continuous independent variables, Lp(a) into (0–100, 100–200, 200+ nmol/l). In the BSCar model score was categorized into three groups (1, 2, 3 - 4), Lp(a) and GGT were modeled using logarithmic transformations, and pulse pressure was modelled as piecewise linear (<49, 49+).

**List of abbreviations**

ABI: ankle brachial index

AIP: atherogenic index of plasma

BSCar: Belcaro score carotid

BSFem: Belcaro score femoral

FIB-4: Fibrosis-4 Liver Index

GGT: gama-glutamyl transferase

HbA1c: glycated hemoglobin

hsCRP: C-reactive protein measured by high sensitivity method

LDL cholesterol: low density cholesterol

Lp(a): lipoprotein (a)

Non-HDL cholesterol: total cholesterol – HDL cholesterol

ORI: Oliva-Roztocil interbranch index

RLPC: remnant plasma cholesterol (total – LDL-HDL cholesterol)

TBI: toe brachial index

T1D: type 1 diabetes

uACR: urine albumin/creatinine ratio

**RESULTS: The associations between vascular parameters and risk factors**

**Ankle brachial index**

| Variable | Estimate | 95% CI | p-value |
| --- | --- | --- | --- |
| (Intercept) | 1.1181 | (1.0839, 1.1523) | <0.0001 |
| Age | 0.0019 | (-0.0026, 0.0064) | 0.0023 |
| Female sex | -0.0475 | (-0.0839, -0.0111) | 0.0009 |
| Connexin37 gene (CC) | 0.0161 | (-0.0338, 0.0660) | 0.5163 |
| Waist circumference if > 85cm | 0.0004 | (-0.0410, 0.0418) | 0.0208 |
| Diabetes duration | 0.0014 | (-0.0326, 0.0354) | 0.0558 |
| GGT (0.3,0.65 ukat/l) | 0.0367 | (-0.0415, 0.1149) | 0.0894 |
| GGT (> 0.65 ukat/l) | 0.0961 | (0.0442, 0.1480) | 0.0264 |
| Pulse pressure if < 55 mm Hg | -0 .0040 | (-0.0190, 0.0110) | 0.0075 |
| Pulse pressure if 55 – 70 mm Hg | -0.0034 | (-0.0077, 0.0009) | 0.0022 |
| Pulse pressure if > 70 mm Hg | -0.0027 | (-0.0080, 0.0026) | 0.0027 |
| Lipoprotein(a) if < 600 nmol/l | 0.0004 | (-0.0489, 0.0497) | 0.0252 |
| Remnant cholesterol | -0.0480 | (-0.0900, -0.0060) | 0.0219 |
| **Interaction with connexin 37 gene polymorphism** | | | |
| GGT(0.3,0.65 ukat/l):Connexin37 gene (CC) | -0.0492 | (-0.1720, 0.0736) | 0.0627 |
| GGT(> 0.65 ukat/l):Connexin37 gene (CC) | -0.1018 | (-0.1647, -0.0389) | 0.0319 |
| Lipoprotein:Connexin37 gene (CC) for Lipoprotein < 600 nmol/l | -0.0004 | (-0.0531, 0.0523) | 0.0268 |
| Remnant cholesterol:Connexin37 gene (CC) | 0.0825 | (0.0240, 0.1410) | 0.0299 |

Adjusted R-squared: 0.1914

**Toe brachial index**

| Variable | Estimate | 95% CI | p-value |
| --- | --- | --- | --- |
| (Intercept) | 1.0978 | (0.9792, 1.2164) | <0.0001 |
| Age | -0.0027 | (-0.0039, -0.0015) | <0.0001 |
| Female sex | -0.2801 | (-0.4924, -0.0678) | 0.0101 |
| Connexin37 gene (CC) | -0.0212 | (-0.0547, 0.0123) | 0.2150 |
| History of dyslipidemia | 0.0194 | (-0.0175, 0.0563) | 0.3035 |
| Pulse pressure | -0.0027 | (-0.0039, -0.0015) | <0.0001 |
| log(Body fat) | 0.0365 | (-0.0039, 0.0769) | 0.0770 |
| AIP | -0.0209 | (-0.0853, 0.0435) | 0.5228 |
| log(Lipoprotein (a)) | -0.0113 | (-0.0203, -0.0023) | 0.0135 |
| C-reactive protein (0.5,1.5 mg/l) | -0.0244 | (-0.0522, 0.0034) | 0.0872 |
| C-reactive protein (1.5,2.5 mg/l) | -0.0128 | (-0.0492, 0.0236) | 0.4908 |
| C-reactive protein (> 2.5 mg/l) | -0.0449 | (-0.0760, -0.0138) | 0.0047 |
| Smoking | 0.0223 | (-0.0105, 0.0551) | 0.1831 |
| **Interaction with connexin 37 gene polymorphism and female sex** | | | |
| History of dyslipidemia:  Connexin37 gene (CC) | -0.0721 | (-0.1241, -0.0201) | 0.0068 |
| log(Body fat):Female sex | 0.0689 | (0.0024, 0.1354) | 0.0421 |
| AIP:Female sex | 0.0950 | (0.0065, 0.1835) | 0.0365 |
| Smoking:Female sex | -0.0644 | (-0.1132, -0.0156) | 0.0100 |
| Female sex:Connexin37 gene (CC) | 0.0443 | (0.0013, 0.0873) | 0.0433 |

Adjusted R-squared: 0.2748

**Belcaro score in carotid arteries**

| Variable | OR (of lower vs higher categories) | 95% CI | p-value |
| --- | --- | --- | --- |
| Age | 0.8279 | (0.8043, 0.8521) | <0.0001 |
| Female sex | 0.4205 | (0.0561, 3.1615) | 0.3270 |
| Connexin37 gene (CC) | 0.1943 | (0.0701, 0.5386) | 0.0064 |
| Waist circumference | 0.9497 | (0.9105, 0.9903) | 0.0158 |
| Diabetes duration | 1.0059 | (0.9782, 1.0345) | 0.6783 |
| Pulse pressure if < 49 mm Hg | 0.9338 | (0.8972, 0.9722) | 0.0008 |
| Pulse pressure if >= 49 mm Hg | 0.9533 | (0.9300, 0.9770) | 0.0004 |
| Body fat | 1.0690 | (0.9981, 1.1451) | 0.0737 |
| AIP | 1.3782 | (0.3415, 5.5592) | 0.6533 |
| Remnant cholesterol | 0.7474 | (0.3157, 1.7691) | 0.5068 |
| HbA1C | 0.9763 | (0.9580, 0.9948) | 0.0108 |
| Smoking | 0.4030 | (0.2361, 0.6874) | 0.0007 |
| **Interaction with connexin 37 gene polymorphism and female sex** | | | |
| Diabetes duration:Female sex | 1.0418 | (0.9964, 1.0896) | 0.0742 |
| Pulse pressure: Female sex for Pulse pressure < 49 mm Hg | 1.0248 | (0.9982, 1.0519) | 0.0636 |
| AIP: Connexin37 gene (CC) | 0.1139 | (0.0141, 0.9236) | 0.0419 |
| Remnant cholesterol:Connexin37 gene (CC) | 6.0574 | (1.4003, 26.2076) | 0.0147 |

**Belcaro score in femoral arteries**

| Variable | OR (of lower vs higher categories) | 95% CI | p-value |
| --- | --- | --- | --- |
| Age | 0.8446 | (0.8143, 0.8758) | <0.0001 |
| Female sex | 0.0322 | (0.0020, 0.5251) | 0.0267 |
| Connexin37 gene (CC) | 5.8115 | (0.7141, 47.3192) | 0.0991 |
| Waist circumference | 0.9098 | (0.8727, 0.9482) | <0.0001 |
| Diabetes duration | 1.0255 | (0.9970, 1.0546) | 0.0824 |
| History of hypertension | 0.6069 | (0.3424, 1.0766) | 0.0877 |
| Body fat | 1.1391 | (1.0550, 1.2299) | 0.0009 |
| Pulse pressure | 0.9782 | (0.9584, 0.9984) | 0.0340 |
| Non-HDL cholesterol | 0.9124 | (0.5890, 1.4129) | 0.6824 |
| AIP | 0.3016 | (0.1553, 0.5856) | 0.0931 |
| GGT | 0.9363 | (0.6306, 1.3912) | 0.7324 |
| HbA1C | 0.9723 | (0.9542, 0.9906) | 0.0024 |
| Smoking | 0.4412 | (0.2601, 0.7483) | 0.0023 |
| **Interaction with connexin 37 gene polymorphism and female sex** | | | |
| Diabetes duration:Female sex | 0.9320 | (0.8888, 0.9780) | 0.0022 |
| Non-HDL cholesterol:Female sex | 2.6315 | (1.3473, 5.1374) | 0.0067 |
| Non-HDL cholesterol:Connexin37 gene (CC) | 0.4776 | (0.2603, 0.8761) | 0.0156 |
| AIP:Female sex | 0.1521 | (0.0178, 1.3025) | 0.0881 |
| GGT:Connexin37 gene (CC) | 2.4308 | (0.8260, 7.1565) | 0.1062 |

**Alb/Crea ratio**

| Variable | Multiplicative effect (exp(coefficient)) | 95% CI | p-value |
| --- | --- | --- | --- |
| (Intercept) | 26.168 | (3.892, 175.264) | 0.0009 |
| Female sex | 3.3467 | (1.798, 6.229) | 0.0001 |
| Connexin37 gene (CC) | 4.1716 | (1.265, 13.760) | 0.0066 |
| Diabetes duration | 1.0244 | (1.0076, 1.0414) | 0.0055 |
| Pulse pressure | 0.9192 | (0.8735, 0.9669) | 0.0010 |
| * (Pulse pressure) ^^2^ | 1.0008 | (1.0004, 1.0012) | <0.0001 |
| Body fat | 0.9528 | (0.9307, 0.9753) | <0.0001 |
| Lipoprotein (a) (100,200 nmol) | 1.5615 | (1.065, 2.290) | 0.0235 |
| Lipoprotein (a) (> 200 nmol/l) | 0.9995 | (0.769, 1.300) | 0.9973 |
| eGDR | 0.7898 | (0.728, 0.857) | <0.0001 |
| Remnant cholesterol if > 0.65 mmol/l | 1.7774 | (1.251, 2.524) | 0.0015 |
| HbA1C | 1.0216 | (1.009, 1.034) | 0.0005 |
| FIB-4 if  < 1.5 | 0.5945 | (0.427, 0.827) | 0.0022 |
| **Interaction with connexin 37 gene polymorphism and female sex** | | | |
| Diabetes duration:Female sex | 0.9655 | (0.943, 0.988) | 0.0023 |
| HbA1c:Connexin37 gene (CC) | 0.9763 | (0.960, 0.992) | 0.0032 |

Adjusted R-squared: 0.3006

Legend:*****quadratic term

**Oliva Roztocil index**

| Variable | Estimate | 95% CI | p-value |
| --- | --- | --- | --- |
| (Intercept) | 0.2608 | (0.2149, 0.3067) | <0.0001 |
| Age | 0.0005 | (0.0002, 0.0008) | 0.0019 |
| Female sex | -0.0653 | (-0.1204, -0.0102) | 0.0207 |
| Connexin37 gene (CC) | -0.0077 | (-0.0221, 0.0067) | 0.2956 |
| * (Diabetes duration)^^2^ | 0.00002 | (0.00001, 0.00003) | 0.0005 |
| History of hypertension | 0.0037 | (-0.0065, 0.0139) | 0.4806 |
| Pulse pressure | 0.0007 | (0.0004, 0.0010) | <0.0001 |
| log(Vitamin D) | -0.0182 | (-0.0284, -0.0080) | 0.0005 |
| Remnant cholesterol | -0.0086 | (-0.0193, 0.0021) | 0.1103 |
| Smoking | 0.0103 | (0.0034, 0.0172) | 0.0037 |
| **Interaction with connexin 37 gene polymorphism and female sex** | | | |
| Hypertension:Connexin37 gene (CC) | 0.0162 | (0.0027, 0.0297) | 0.0204 |
| log(Vitamin D):Female sex | 0.0214 | (0.0072, 0.0356) | 0.0030 |
| Remnant cholesterol:Connexin37 gene (CC) | 0.0205 | (0.0031, 0.0379) | 0.0212 |
| Female sex:Connexin37 gene (CC) | -0.0141 | (-0.0278, -0.0004) | 0.0457 |

Adjusted R-squared: 0.3306

Legend:*****quadratic term

**List of abbreviations**

AIP: atherogenic index of plasma

Cx37: connexin 37 – CC – CC homozygotes

eGDR: estimated glucose disposal rate

FIB-4: fibrosis-4 liver index

GGT: gamma-glutamyl transferase

HbA1c: glycated hemoglobin

hsCRP: C-reactive protein measured by the high-sensitivity method

LDL cholesterol: low-density cholesterol

Lp(a): lipoprotein (a)

Non-HDL cholesterol: total cholesterol – HDL cholesterol

RLPC: remnant plasma cholesterol (total – LDL-HDL cholesterol)
